# Location matters: highly divergent protein levels in samples from different CNS compartments in a clinical trial of rituximab for progressive MS

**DOI:** 10.1186/s12987-020-00205-4

**Published:** 2020-07-29

**Authors:** Joakim Bergman, Anders Svenningsson, Per Liv, Tommy Bergenheim, Joachim Burman

**Affiliations:** 1 Department of Clinical Sciences, Umeå University, 901 85 Umeå, Sweden; 2 Department of Clinical Sciences, Karolinska Institutet Danderyd Hospital, Stockholm, Sweden; 3 Department of Public Health and Clinical Medicine, Umeå University, Umeå, Sweden; 4 Department of Neurosciences, Uppsala University, Uppsala, Sweden

**Keywords:** Cerebrospinal fluid, Microdialysis, Ventricular CSF, Lumbar CSF, Interstitial fluid, CSF compartments, CSF proteins, Progressive MS

## Abstract

**Background:**

The relationship between proteins in different CNS extracellular compartments is unknown. In this study the levels of selected proteins in three compartments in people with progressive multiple sclerosis (PMS) were compared.

**Methods:**

During an open label, phase 1b study on intraventricular administration of rituximab for PMS, samples were collected from the interstitial space (ISS) of the brain through microdialysis. Samples were also obtained from ventricular and lumbar cerebrospinal fluid (CSF). These samples were analyzed with a multiplexed proximity extension assay, measuring the levels of 180 proteins split equally between two panels, detecting proteins associated with immunology and neurology, respectively.

**Results:**

Considerable differences in concentrations were observed between the three analyzed compartments. Compared to ventricular CSF, ISS fluid contained statistically significant higher levels of 25 proteins (84% immunology panel and 16% neurology panel). Ventricular CSF contained significantly higher levels of 54 proteins (31% immunology panel and 69% neurology panel) compared to ISS fluid, and 17 proteins (76% immunology panel and 24% neurology panel) compared to lumbar CSF. Lumbar CSF showed significantly higher levels of 115 proteins (32% immunology panel and 68% neurology panel) compared to ventricular CSF. The three compartments displayed poor correlation with a median Spearman’s rho of -0.1 (IQR 0.4) between ISS and ventricular CSF and 0.3 (IQR 0.4) between ventricular and lumbar CSF.

**Conclusion:**

A substantial heterogeneity in the protein levels of samples obtained from different CNS compartments was seen. Therefore, data obtained from analysis of lumbar CSF should be interpreted with caution when making conclusions about pathophysiological processes in brain tissue.

## Background

The study of in vivo processes and pathophysiological mechanisms in the central nervous system (CNS) of humans poses many challenges. Brain biopsies are not readily available and, where possible, tissue samples processed ex vivo will inevitably suffer from artefacts due to the rapid loss of viability. Imaging studies are limited for translating structure to function, particularly on the cellular or subcellular level. In vitro models still have many challenges to overcome [1].

One of the most common methods to study in vivo processes in the CNS is the biochemical analysis of cerebrospinal fluid (CSF). The concept of the “third circulation” suggesting that CSF flows through the ventricles, cisterns and subarachnoid space and is reabsorbed into the blood at the arachnoid villi, was introduced by Cushing already in 1925 [2]. It has been assumed that CSF samples obtained by lumbar puncture are related to pathophysiological processes in the CNS. This has been utilized for diagnostic purposes as well as for markers of disease progression, e.g. oligoclonal bands in support of the diagnosis of multiple sclerosis and neurofilament-light as an indicator of axonal damage in various neurological diseases [3]. However, to which extent lumbar CSF accurately reflects ongoing processes in the brain is presently unknown.

In an open label, phase 1b study on intraventricular administration of rituximab for progressive multiple sclerosis (PMS), the study design included measurements of protein levels in three different CNS compartments: the interstitial space (ISS) of the brain, the right lateral ventricle and the lumbar CSF space. This enabled us to determine to what degree the composition of proteins was similar between compartments.

## Materials and methods

### Ethical approval of standard protocols, registrations, and patient consent

Samples were collected in the multicenter, prospective, open-label, phase 1b Intrathecal Treatment Trial in Progressive Multiple Sclerosis (ITT-PMS) [4] and the related extension study. Both trials were observational with no control groups and no randomization of patients. The trials were approved by the Regional Ethics Review Board in Umeå and registered in the EU Clinical Trial Register (EudraCT 2008-002626-11 and 2012-000721-53). All participating subjects provided written informed consent.

### Study cohort

Subjects were recruited from the outpatient clinics of the Departments of Neurology at two Swedish university hospitals (Umeå and Uppsala). Individuals with primary (n = 8) or secondary (n = 15) PMS were recruited between June 27, 2009, and May 11, 2015. In total there were 16 women and 7 men, their mean age 46 ± 9 years, with an Expanded Disability Status Scale (EDSS) between 4.0 and 7.0. Of the 23 participants in ITT-PMS, 19 accepted to participate in the extension study. In total 16 individuals from the ITT-PMS and its extension study provided samples for this study.

### Surgical procedure

Under general anesthesia, a ventricular catheter was implanted into the right frontal horn and connected to a subcutaneous Ommaya reservoir, as previously described [4]. In order to measure levels of free, unbound analyte concentrations in the extracellular fluid in brain tissue a microdialysis catheter was inserted in 10 patients [5]. The catheter was placed in parallel to the ventricular catheter and with the semipermeable membrane located in the periventricular white matter. The localization was confirmed by a CT scan performed within a couple of hours after the MD catheter was implanted.

### Microdialysis

The brain catheter had a semipermeable membrane that was 10 mm long and had a pore size of 100 kDa (CMA71; CMA Microdialysis, Stockholm, Sweden). The catheter was perfused with an aqueous solution that closely resembles the ionic composition of the surrounding tissue fluid with the addition of Dextran (30 g Dextran-60 1000 mL^−1^) (Plasmodex^®^) to prevent microfiltration. The catheter was connected to a 2.5 mL syringe placed in a micro infusion pump with a flow rate of 1.0 μL/min (CMA 107; CMA Microdialysis, Stockholm, Sweden).

### Investigational drug

The investigational drug in ITT-PMS and the related extension study was rituximab. In the ITT-PMS, rituximab was administrated in the Ommaya reservoir as three 25 mg intraventricular injections 1 week apart. The first injection was administered 5 days after the Ommaya reservoir was implanted. In the extension study, one single dose of 25 mg rituximab was given every sixth months; the first treatment was given at the same time-point as the last clinical follow-up in the ITT-PMS trial, and the last treatment was given 6 months before the end of the extension study.

### Sample collection

In those patients that received a microdialysis (MD) catheter, samples were collected from the MD catheters throughout the first week after implantation. Samples were collected continuously, and sample vials were switched six times each day; three times at 6-h intervals in the daytime, and three times at 2-h intervals during the night. Collected samples were immediately stored at − 80 °C. The first sample collected between zero and 6 h after implantation was used for the analyses in this study.

At surgery, 2 mL ventricular CSF was collected through aspiration of the Ommaya reservoir. Before each rituximab administration, 5 mL ventricular CSF was aspirated from the Ommaya reservoir. In the ITT-PMS study, lumbar puncture of the subarachnoid space was made before surgery and at each follow-up, when 10 mL lumbar CSF was collected by passive flow. CSF pressure was not measured in either instance. The present study included 13 paired samples of lumbar and ventricular CSF obtained at the same follow-up visit. Paired samples were obtained at the 12 months follow-up visit from six patients in the original ITT-PMS study, from one patient at the 12 months follow-up visit in the extension study and from six patients at the 24 months follow-up visit in the extension study. Some patients did not enter the extension study directly and therefore these samples were obtained between 12 and 60 months after the Ommaya reservoir was implanted. Earlier samples were not included to minimize possible effects of the surgical procedure on the protein levels. All CSF samples were snap frozen at − 80 °C in 1 mL aliquots stored in polypropylene tubes and handled according to consensus guidelines [6].

**Fig. 1:**
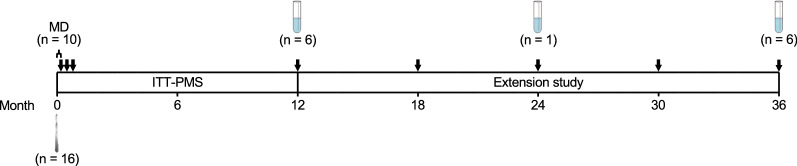
Design of the studies displaying treatments given and sample collections used in this paper. The bar shows the transition from the Intrathecal Treatment Trial in Progressive Multiple Sclerosis (ITT-PMS) to the follow-up extension study, with time shown below in months. Black arrows display points of treatment. Scalpel indicates implantation of Ommaya reservoir and collection of ventricular CSF during surgery, and test tubes indicate collection of paired lumbar and ventricular CSF. MD, microdialysis; n, number of participants

The location and timing of the samples used for the study are summarized in Fig. 1.

### Protein analysis

The samples were analyzed with a proximity extension assay (PEA) through a service provided by the manufacturer (Olink Proteomics, Uppsala, Sweden). PEA is a multiplex immunoassay which enables measurement of up to 96 proteins simultaneously from small sample volumes. Pairs of oligonucleotide-labelled antibodies directed towards a target protein in the sample are used. Each of the 96 oligonucleotide antibody-pairs contains unique DNA sequences allowing hybridization only to each other. Subsequent proximity extension will create 96 unique DNA sequences which are amplified by real-time PCR and then quantified (Additional file 1: Figure S1) [7]. The method is semi-quantitative and estimates the amount of each protein based on the number of PCR cycles needed for detection. This was then used as base for pair-wise comparisons between compartments and time-points. Extensive information about the PEA is available on the manufacturer’s web site (https://www.olink.com).

Two fixed panels detecting 91 proteins each were used. One, the *inflammation* panel, analyzed new and established human proteins related to inflammation. The other, the *neurology* panel, analyzed a mix of established proteins related to neurobiological processes (e.g. neural development, axon guidance, synaptic function), as well as some more exploratory proteins with broader roles in processes such as cellular regulation, immunology, development and metabolism. There was an overlap of two proteins, and in total 180 unique proteins were analyzed. The proteins had a molecular weight between 10 and 232 kDa. Reference values, limit of detection, lower limit of quantification, upper limit of quantification, intra and inter assay CV for each analyte is available at the manufacturer’s web site (https://www.olink.com).

### Statistical analysis

The statistical software R version 3.5.3 was used for all analyses. Differences in protein levels between locations and between time points were investigated using two-sided Wilcoxon signed-rank tests. If a difference between two groups were detected with this test, the group with higher values was said to be ‘higher’ and the group with lower values was said to be ‘lower’. Correlations between protein levels were estimated using Spearman correlation coefficients. Protein levels below limit of detection (LOD) were set to be equal to LOD/2, motivated by the use of non-parametric rank-based statistical analyses. Standardized mean differences between locations and time points were calculated as the ratio of difference in means and standard deviation. A significance level of 0.05 was employed with no correction for multiple comparisons.

## Results

### Participants

Of the 23 participants in the main study, 13 had suitable paired CSF samples and 10 had partaken in MD. In total, due to overlap, this resulted in 16 individual participants.

In the subgroup with paired samples, 10 were females and 3 males. Mean age at disease onset was 32.7 (SD 11.8) years and mean age at inclusion was 47.4 (SD 8.7) years (mean disease duration at inclusion was 14.6 (SD 9.2) years). PPMS was diagnosed in 38% (n = 5) of participants, while 62% (n = 8) had a diagnosis of SPMS. Median EDSS score at inclusion was 6.5 (IQR 0.5).

In the MD subgroup 8 were females and 2 males. Mean age at disease onset was 33.7 (SD 11.9) years and mean age at inclusion was 46.0 (SD 7.9) years (mean disease duration at inclusion was 12.2 (SD 10.0) years). PPMS was diagnosed in 60% (n = 6) of participants, while 40% (n = 4) had a diagnosis of SPMS. Median EDSS score at inclusion was 5.5 (IQR 1.9).

### Quality control

The samples were run on four different plates with a control sample run in duplicate on each plate. The pooled intra assay CV was 2.4–11% and the pooled inter assay CV was 7.6–10%.

### Comparison between MD samples and ventricular CSF obtained at surgery

**Fig. 2:**
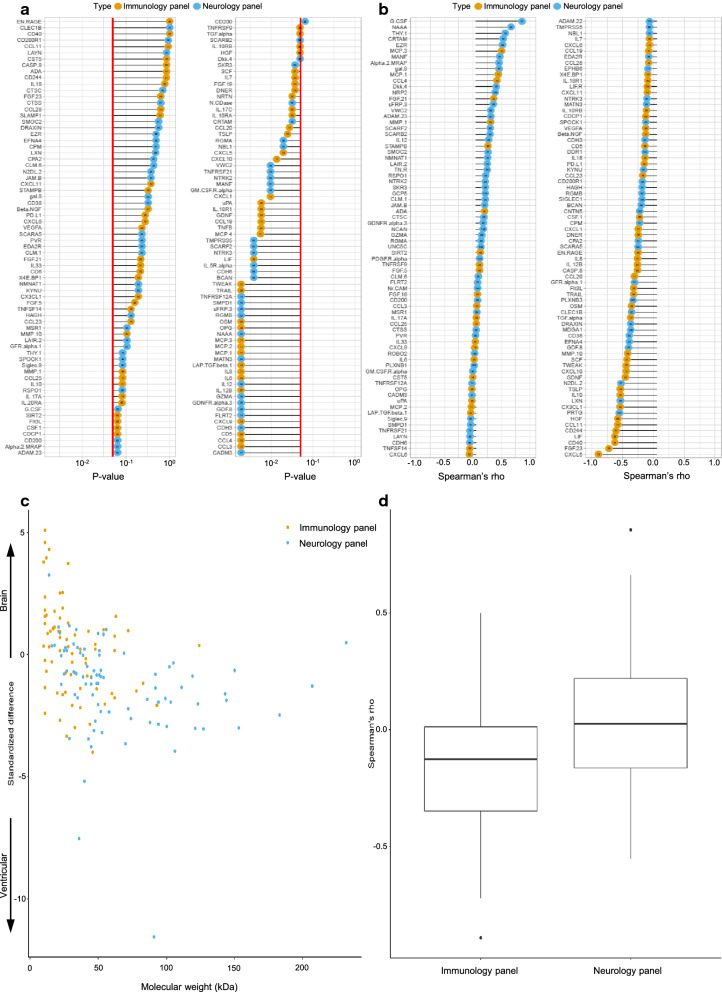
Comparison of proteins detected between extracellular fluid (ECF) from brain and ventricular CSF at the time of surgery (**a**) with p-values listed for all detected proteins and 0.05 indicated by red line. **b** Spearman’s rho showing correlation for all detected proteins with lines starting at zero, indicating positive and negative correlation by right and left, respectively. **a**, **b** Circles in orange mark proteins from the immunological panel, and circles in blue mark proteins from the neurological panel. Molecular weights (kDa) are shown by numbers within coloured circles. **c** Spread of proteins between compared compartments based on molecular weight (kDa), where a positive standardized difference indicates higher levels in ISS samples from brain and a negative standardized difference indicates higher levels in ventricular CSF samples. **d** Boxplot for spread of spearman’s rho subdivided according to the two protein panels

Out of the 180 analyzed proteins, 157 could be detected in at least one of these two compartments. Of these, 79 were different with statistical significance between the two compartments; 32% (n = 25) were higher in MD samples, and 68% (n = 54) were higher in ventricular CSF samples. Of the 25 proteins that were higher in MD samples 84% (n = 21) came from the immunology panel and 16% (n = 4) from the neurology panel. Of the 54 proteins that were higher in ventricular CSF samples, 31% (n = 17) came from the immunology panel and 69% (n = 37) from the neurology panel. The median Spearman’s rho was − 0.1 (IQR 0.4), essentially demonstrating that there was no correlation between the proteins in these two compartments (Fig. 2).

### Comparison between lumbar and ventricular CSF samples obtained at follow-up

**Fig. 3:**
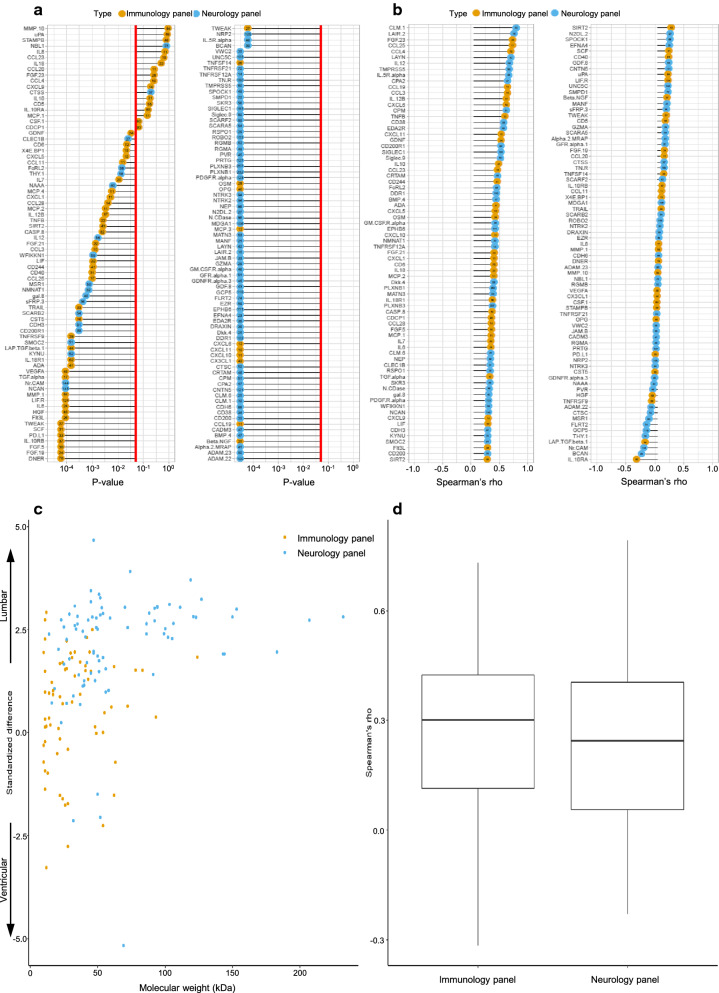
Comparison of proteins detected between lumbar CSF and ventricular CSF, obtained at follow-up (between 12 and 60 months post-surgery). **a** p-values listed for all detected proteins with 0.05 indicated by red line. **b** Spearman’s rho showing correlation for all detected proteins with lines starting at zero, indicating positive and negative correlation by right and left, respectively. **a**, **b** Circles in orange mark proteins from the immunological panel, and circles in blue mark proteins from the neurological panel. Molecular weights (kDa) shown by numbers within coloured circles. **c** Spread of proteins between compared compartments based on molecular weight (kDa), where a positive standardized difference indicates higher levels in lumbar CSF samples and a negative standardized difference indicates higher levels in ventricular CSF samples. **d** Boxplot for spread of spearman’s rho subdivided according to the two protein panels

Out of 180 analyzed proteins, 150 could be detected in at least one of the two compartments. Of these, 132 were different with statistical significance between the two compartments; 87% (n = 115) were higher in lumbar CSF samples, and 13% (n = 17) were higher in ventricular CSF samples. Of the 115 proteins that were higher in lumbar CSF samples, 32% (n = 37) came from the immunology panel and 68% (n = 78) from the neurology panel. Of the 17 proteins that were higher in ventricular CSF samples, 76% (n = 13) came from the immunology panel and 24% (n = 4) from the neurology panel. The median Spearman’s rho across all proteins was 0.3 (IQR 0.4), displaying a poor correlation between the two compartments in general. Molecular weight did not correlate with location in either compartment (Fig. 3).

### Comparison between ventricular CSF obtained at surgery and follow-up

**Fig. 4:**
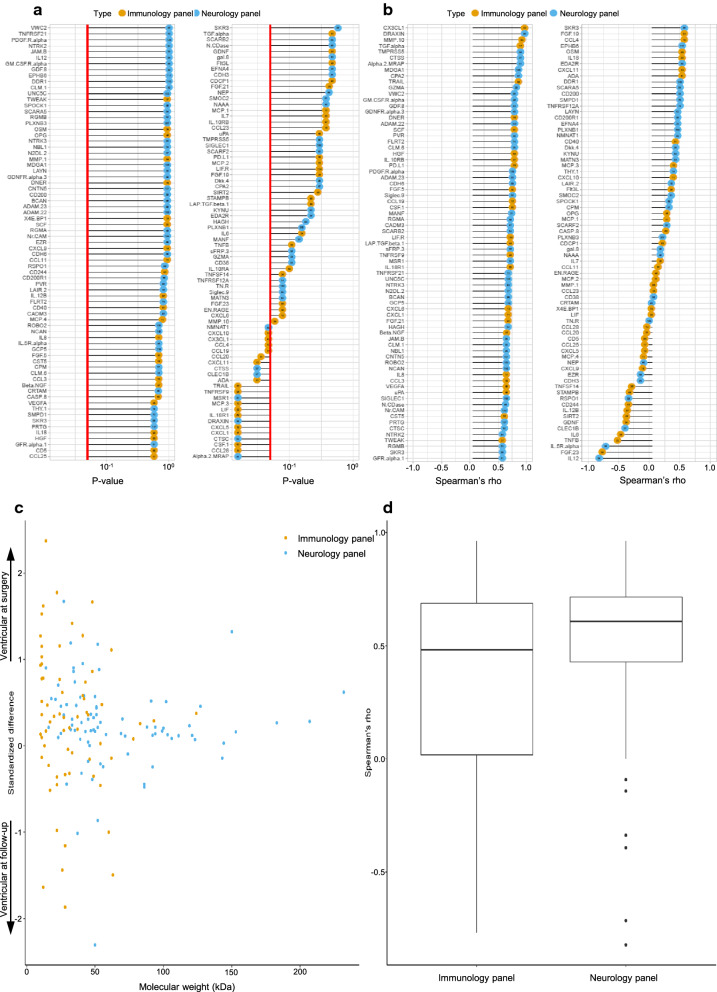
Comparison of proteins detected between ventricular CSF at surgery and ventricular CSF obtained at follow-up (between 12 and 60 months post-surgery). **a** p-values listed for all detected proteins with 0.05 indicated by red line. **b** Spearman’s rho showing correlation for all detected proteins with lines starting at zero, indicating positive and negative correlation by right and left, respectively. **a**, **b** Circles in orange mark proteins from the immunological panel, and circles in blue mark proteins from the neurological panel. Molecular weights (kDa) shown by numbers within coloured circles. **c** Spread of proteins between compared compartments based on molecular weight (kDa), where a positive standardized difference indicates levels at surgery and a negative standardized difference indicates levels at follow-up. **d** Boxplot for spread of spearman’s rho subdivided according to the two protein panels

Out of the 180 analyzed proteins, 146 could be detected in samples from at least one of the two timepoints. Of these, 123 (84%) did not differ significantly between the two timepoints. Of the 23 proteins that differed with statistical significance between the timepoints; 78% (n = 18) of the proteins were higher in samples obtained at surgery and 22% (n = 5) in samples obtained at follow-up. Of the 18 proteins that were higher in samples obtained at surgery, 72% (n = 13) came from the immunology panel and 28% (n = 5) from the neurology panel. Of the 5 proteins that were higher in samples obtained at follow-up 60% (n = 3) came from the immunology panel and 40% (n = 2) from the neurology panel. The median Spearman’s rho for proteins in the immunology panel was 0.5 (IQR 0.7), indicating a slight correlation. The median Spearman’s rho for proteins in the neurology panel was 0.6 (IQR 0.3), indicating an overall moderate correlation. Molecular weight did not correlate with location at either timepoint (Fig. 4).

## Discussion

In this study we compared the levels of 180 proteins in three different CNS compartments. Our main finding was that there was a strikingly poor correlation between the protein levels of all compartments, for the most part not more than expected by chance. Consequently, a model based on a gradual shift of proteins from the ISS fluid to the ventricles and further on into the lumbar cistern gained no support. On the other hand, we demonstrated a moderate correlation between the protein levels in samples obtained from the same location but at different timepoints, suggesting some degree of individual stability over time in each compartment.

According to mainstream theory, CSF is chiefly formed by the choroid plexus, based upon the historical canine experiments of Dandy [8]. Then, CSF moves through a unidirectional flow through the ventricles, cisterns and subarachnoid space and is finally reabsorbed into the blood at the site of the arachnoid villi [2]. This theory is likely an oversimplification and there are good reasons to believe that CSF circulation is much more complex: a combination of directed bulk flow, pulsatile movements, and continuous bi-directional fluid exchange at the blood brain barrier and the cell membranes at the borders between CSF and ISS spaces probably all contribute to the movement and composition of CSF [9]. Furthermore, the recent discoveries of the glymphatic system and meningeal lymphatic vessels [10–13] have provided novel insights on the clearance of proteins and metabolites. Our data are more in line with this complex model and highlight the importance of not extrapolating data obtained from one compartment in the CNS to another.

As a first objective we wanted to establish whether the protein composition of the ISS was similar to CSF from the right lateral ventricle. Most of the proteins in our panels could be detected by the PEA, but the Spearman’s rho was centered around zero, with an equal distribution on both sides, suggesting that there was no meaningful correlation between these two compartments. Also, the distribution of the levels of the proteins was fairly equal with 36% of proteins being higher in the ISS and 64% higher in ventricular CSF samples, again suggesting a random effect. However, the results are likely to be influenced by the pore size (100 kDa) of the semipermeable membrane of the MD catheter, causing a reduced recovery of proteins starting at 40–50 kDa in size. Recovery by MD also depends on the individual characteristics of the analyzed proteins, which influence physical passage through the membrane as well as adhesion to the membrane and tubes. Absolute and relative recovery can be enhanced by adding dextran to the MD fluid and the flow rate was chosen in order to optimize the absolute recovery volume in order to facilitate the laboratory analyses [14, 15]. Adhesion of the proteins to the tubes is highest in the first 5–10 min after the system is set up. After that the inner lining of the tubes will be saturated with proteins and recovery will stabilize [15]. Since the MD fluid analyzed in this study was collected during 6 h we believe that the issue of loss of recovery due to adhesion is of minor importance.

Most larger proteins analyzed in ISS will probably have a recovery of < 40%, suggesting that the actual concentrations of proteins in ISS are higher than observed here, which may have led to an overestimation of the difference in protein levels. However, this is less likely to influence the over-representation of proteins from the immunology panel in the ISS, since they have a low molecular weight with a higher expected recovery. Despite the issue of recovery, a number of proteins showed a higher level in ISS compared to ventricular CSF, which strongly suggests a true difference for those particular proteins.

When using dextran, there is always a possibility of dextran leakage from the catheter to the extracellular space, which may influence the local microenvironment around the catheter. For Dextran-70 a histological inflammation reaction has been demonstrated but not for Dextran-500 [16]. Unfortunately, no analysis of the composition of proteins in the extracellular space was done in that study. To our knowledge, no such study has been performed with Dextran-60. Due to the inherent nature of MD, great precaution has to be taken when discussing the absolute levels of proteins obtained by MD. Nevertheless, MD is currently the best method to obtain samples from extracellular space allowing non-targeted mass analysis of protein spectra. Despite the limitations of the technique we believe that our findings lead to the conclusion that it is unlikely that the ISS of the brain directly communicates with the CSF of the ventricles. A more reasonable conclusion is perhaps that these proteins are cleared to the perivenous space and then drained out of brain into the cervical lymphatic system [17, 18].

As a second objective, we wanted to establish whether the protein composition of the CSF from the right lateral ventricle was similar to the CSF in the lumbar subarachnoid space. It has been demonstrated that lumbar injection of radionuclides [19] as well as gadolinium contrast agents [20] do not enter the ventricular system under normal conditions, which supports a caudally directed flow of CSF from the ventricles to the lumbar region. A majority of the proteins in our study displayed higher levels in the lumbar cistern. Furthermore, our data suggested a very poor correlation between the protein levels in the two compartments. These data highlight the complex relationship between different subcompartments of the total CSF compartment including the various ways proteins enter CSF through the blood-brain-barrier (BBB), blood-spinal cord barrier (BSCB) and blood-CSF-barrier (BCB) [21, 22]. For immunological substances, an explanation may be that several of these have their origins in the leptomeninges. Proteins synthesized in the leptomeninges have indeed been shown to be highly enriched in the lumbar compartment compared with the ventricles [23]. For proteins in the neurology panel, the finding of substantially higher concentrations in the lumbar compared with ventricular CSF is more intriguing since the opposite relation has been reported for brain cell-derived structural proteins [23]. One possible explanation to this observation could be that some of the proteins in the neurology panel were more abundantly expressed in the spinal cord, from which the contribution of specific protein content to the lumbar CSF is likely to be greater than from the brain. In addition, the overall rostral—caudal flow of CSF with only around 20% of the total volume enclosed in the ventricles could lead to a slower bulk flow with an accumulation of content in the larger lumbar cistern.

The strongest correlation between compartments in this study was between proteins obtained from the same (ventricular) compartment but at different timepoints. In lumbar CSF, the most common proteins have been extensively studied and rather narrow reference ranges have been established [24, 25], indicating the same type of stability over time within this sub-compartment as well. These observations are further reinforced by a recent study, in which it was shown that the intraindividual variation of the proteome for lumbar CSF was considerably more stable over time than the interindividual variations between different individuals [26]. In addition, previous investigators have demonstrated a small but consistent concentration gradient within the lumbar compartment when CSF was withdrawn in 4 mL portions up to 24 mL CSF [27]. Altogether, these data are consistent with a CSF compartment that can be further divided into at least two sub-compartments (i.e. lateral ventricles and lumbar sac), each with a rather stable composition but a complex and variable exchange of specific proteins.

**Fig. 5:**
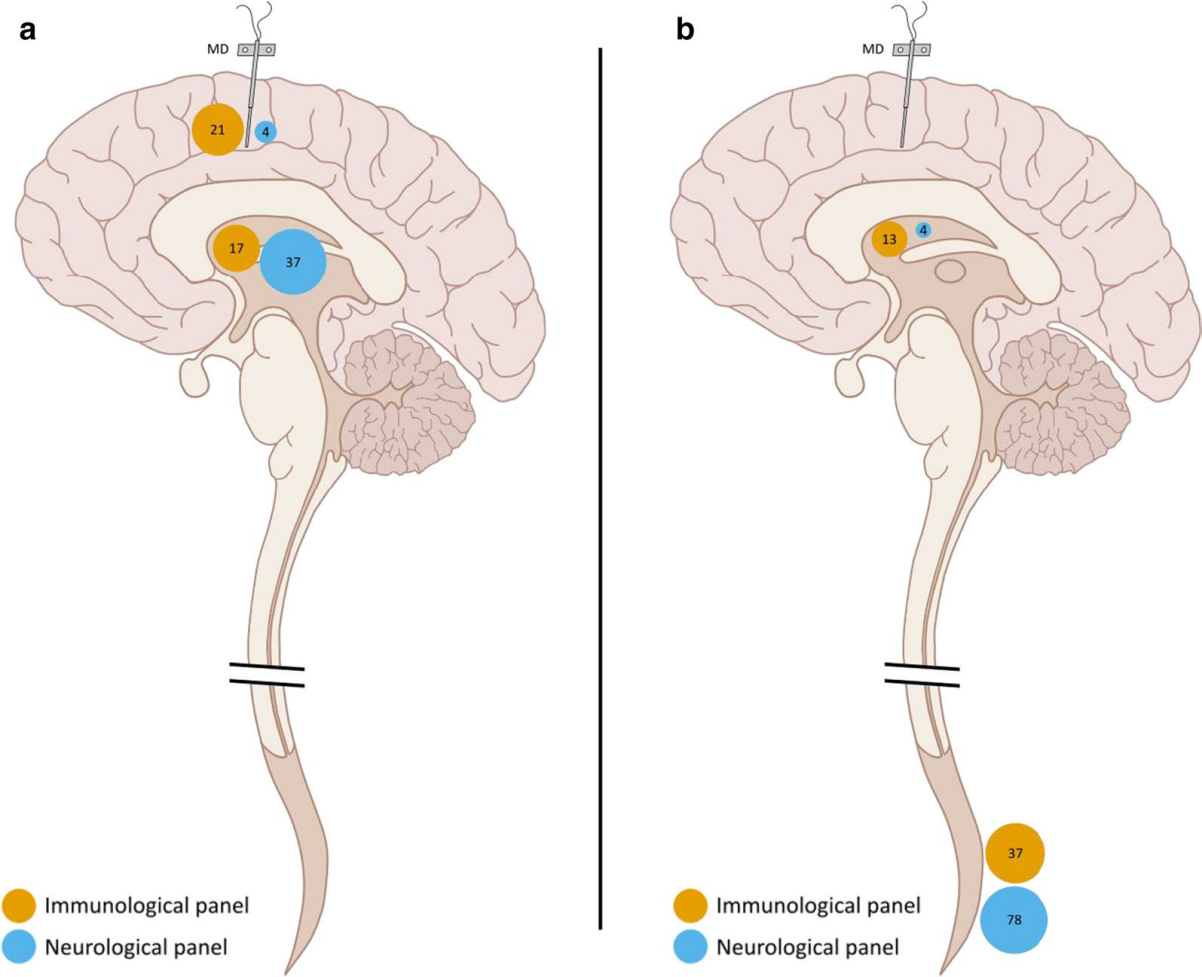
Overview of the analysed compartments indicating the number of proteins found showing the higher levels at each site. The size of the circles indicates the percentage of protein found from their respective panel. Numbers within circles show absolute number of proteins. **a** Comparing samples from interstitial space (ISS) with ventricular CSF. **b** Comparing ventricular and lumbar CSF. MD, microdialysis

Analyses of different proteins in body fluids can be done for several purposes where the specific purposes are linked to specific and sometimes different requirements. To establish a biomarker, the protein needs to be statistically associated with a clinical outcome, but knowledge of the actual role of the protein in disease is strictly not necessary. On the other hand, in order to study a pathophysiological disease process, the sampling site needs to some extent mirror the anatomical site where the pathological process occurs, in order to draw valid conclusions from observations. Up to 80% of the proteins in the CSF are believed to originate from the blood, leaving only a fraction of CNS-specific content mirroring disease processes within the CNS [23]. An obstacle to the research of human CNS disorders is the difficulty in obtaining representative tissue samples from living patients with non-malignant disease. Cerebrospinal fluid collected at lumbar puncture has often been regarded as a window to the CNS. In some diseases, CSF findings have contributed to the unfolding of pathological processes in the brain and spinal cord, one example being the dementia field [28]. In those neurodegenerative disorders, the CSF proteins that panned out to be of significance were all structural proteins of the CNS, similar to those in the *neurology* panel of our study. The most important biomarker of structural damage to the CNS in MS is neurofilament light protein (NFL), which was not a part of the *neurology* panel used here. NFL is an extraordinarily stable protein [29], which slowly diffuses from the site of injury to the CSF compartment [3]. NFL is believed to be accumulated in the lumbar sac, increasing the sensitivity of this biomarker when obtained at this location [23]. The opposite is to be expected when studying paracrine cytokines or chemokines, which are locally produced in the tissue. These are likely to degrade over time and distance from the site of production. Proteins from the *immunology* panel in this study were more abundant in the ISS compared with ventricular CSF while the opposite was the case between ventricular and lumbar CSF indicating a complex relationship between the different CNS compartments for these substances (Fig. 5, Additional file 2: Table S1).

Our study had several weaknesses, the major one being the small sample size (n = 10 for paired MD/ventricular-CSF and n = 13 for paired ventricular-CSF/lumbar-CSF samples). The study was planned as an exploratory part of a clinical trial with primary endpoint safety and feasibility, which by its phase 1b nature was limited in terms of patient numbers [4]. The invasive neurosurgical procedures used in the study put a clear limit on patient recruitment. The sampling itself could potentially influence the pressure dynamics in the CSF compartments, which might affect synthesis and/or distribution of some brain-derived proteins. The most common adverse event related to intraventricular injection was a short-lasting vertigo, which we believe was created by a disturbance of pressure equilibrium [4]. However, since collection of samples were done before injection, we believe it is unlikely that this affected the protein concentrations in the samples used for this study.

We decided to adopt a broad approach to the analysis of constituents in the different CNS compartments. The large number of tested proteins in relation to the small number of patients obviously prevents reliable conclusions regarding specific proteins and their role in PMS, although further hypotheses on specific proteins could be formulated and tested in future studies. P-values from a wide explanatory analysis, such as from the present study, cannot be interpreted in the same way as for a confirmatory analysis. Corrections for multiple comparisons to control for family wise error or false discovery rate was deemed not meaningful due to the loss of power it would imply. However, it should be noted that the expected number of type 1-errors when testing 180 proteins is 9, assuming no real differences exist and that we obtained more statistically significant differences in all performed compartment comparisons. Another weakness is that we were not able to make any comparison between protein levels in the ISS and lumbar CSF from samples obtained at the very same timepoint, intuitively perhaps the most interesting. However, the design of the study, with a baseline lumbar puncture performed at a timepoint preceding the surgical procedure with a substantial variability in time, would have made a comparison difficult to interpret. In contrast, the other comparisons between different compartments were done on the samples obtained more or less simultaneously (at a most within a few hours) in the compared compartments. However, the overall lack of correlation between the other investigated compartments in this study makes it unlikely that protein concentrations obtained in lumbar CSF would reliably reflect the composition of proteins pertaining to focal pathology in the brain tissue. This should be confirmed in future studies, by obtaining simultaneous samples from ISS and lumbar CSF and analyze the relationship between key inflammatory substances in these compartments.

## Conclusions

The results from this study indicate a substantial heterogeneity regarding the proteins within different compartments of the CNS. Therefore, great care should be taken when analyzing data obtained from analysis of lumbar CSF samples in relation to the underlying pathological processes in the CNS tissue. This seems to be particularly pertinent to the interpretation of levels and patterns of inflammatory substances.

## Supplementary information

**Additional file 1: Figure S1.** Proximity Extension Assay (PEA). (A) a matched pair of antibodies linked to unique oligonucleotides are added for each protein of interest. (B) When bound to a protein, the paired oligonucleotides come in close vicinity of each other and hybridize, which enables a DNA amplification by the addition of a DNA polymerase. (C) The DNA amplification is detected and quantified by quantitative real-time PCR. Picture provided by Olink Proteomics.

**Additional file 2: Table S1.** Overview of results. Left side of the table lists all proteins detected in the indicated compartment, while the right side of the table lists the number of proteins with a *p*-value ≤ 0.05. The numbers show the amount of proteins with a higher level in respective compartment.

## Data Availability

The study protocols of the ITT-PMS and extension study are available on request from the Principal Investigator (Svenningsson A.). By Swedish law, data from clinical trials are archived by the Principal Investigator for a minimum of 10 years. Raw data can be made available within this time frame in a de-identified form upon written request to the Principal Investigator under condition that additional ethical approval is obtained from the Ethical Review Board.

## Abbreviations

CNS: Central nervous system
CSF: Cerebrospinal fluid
EDSS: Expanded disability status score
ISS: Interstitial space
LOD: Limit of detection
MD: Microdialysis
MS: Multiple sclerosis
NFL: Neurofilament light
PEA: Proximity extension assay
PMS: Progressive multiple sclerosis
PPMS: Primary progressive MS
SPMS: Secondary progressive MS
