# Supplementary figures and images for: Location matters: highly divergent protein levels in samples from different CNS compartments in a clinical trial of rituximab for progressive MS
Source: Fluids Barriers CNS. 2020 Jul 29;17:49. doi: 10.1186/s12987-020-00205-4 (PMC7390226; doi:10.1186/s12987-020-00205-4)

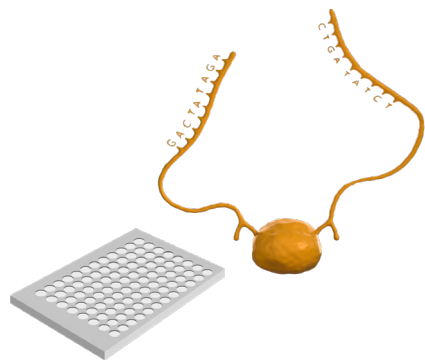

(A) Incubation

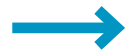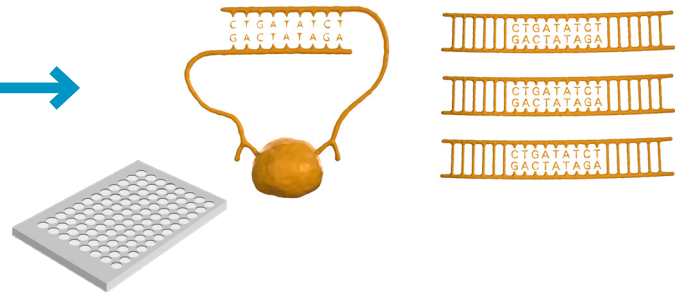

(B) Extension and  
pre-amplification

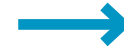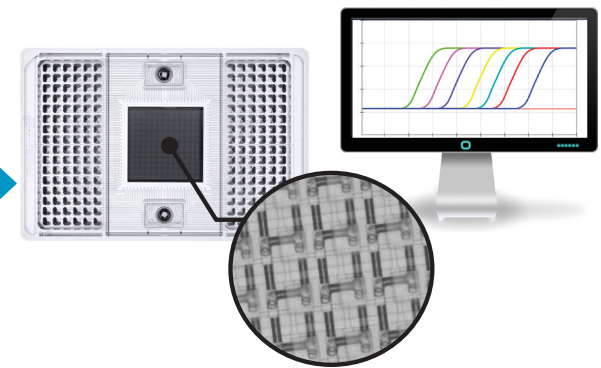

(C) Detection

Supplement: Supplementary file 1 — Additional file 1: Figure S1. Proximity Extension Assay (PEA). (A) a matched pair of antibodies linked to unique oligonucleotides are added for each protein of interest. (B) When bound to a protein, the paired oligonucleotides come in close vicinity of each other and hybridize, which enables a DNA amplification by the addition of a DNA polymerase. (C) The DNA amplification is detected and quantified by quantitative real-time PCR. Picture provided by Olink Proteomics. [file 12987_2020_205_MOESM1_ESM.pdf]
