# Supplementary material for: Location matters: highly divergent protein levels in samples from different CNS compartments in a clinical trial of rituximab for progressive MS
Source: Fluids Barriers CNS. 2020 Jul 29;17:49. doi: 10.1186/s12987-020-00205-4 (PMC7390226; doi:10.1186/s12987-020-00205-4)
Supplement: Supplementary file 2 — Additional file 2: Table S1. Overview of results. Left side of the table lists all proteins detected in the indicated compartment, while the right side of the table lists the number of proteins with a p-value ≤ 0.05. The numbers show the amount of proteins with a higher level in respective compartment. [file 12987_2020_205_MOESM2_ESM.docx]

|  | | | | | | |
| --- | --- | --- | --- | --- | --- | --- |
|  | All proteins found | | | Proteins found with *p* ≤ 0.05 | | |
|  | Total | Immunology panel | Neurology panel | Total | Immunology panel | Neurology panel |
| MD and ventricular CSF obtained at surgery, n (%) | 157 (-) | 74 (47) | 83 (53) | 79 (-) | 38 (48) | 41 (52) |
| MD, n (%) | 64 (41) | 44 (69) | 20 (31) | 25 (32) | 21 (84) | 4 (16) |
| Ventricular, n (%) | 93 (59) | 30 (32) | 63 (68) | 54 (68) | 17 (31) | 37 (69) |
| Spearman’s rho, median (IQR) | -0.1 (0.4) | -0.1 (0.4) | 0.0 (0.4) | - | - | - |
|  |  |  |  |  |  |  |
| Lumbar and ventricular CSF at follow-up, n (%) | 150 (-) | 66 (44) | 84 (56) | 132 (-) | 50 (38) | 82 (62) |
| Lumbar, n (%) | 127 (85) | 47 (37) | 80 (63) | 115 (87) | 37 (32) | 78 (68) |
| Ventricular, n (%) | 23 (15) | 19 (83) | 4 (17) | 17 (13) | 13 (76) | 4 (24) |
| Spearman’s rho, median (IQR) | 0.3 (0.4) | 0.3 (0.3) | 0.2 (0.3) | - | - | - |
|  |  |  |  |  |  |  |
| Ventricular CSF at surgery and at follow-up, n (%) | 146 (-) | 65 (45) | 81 (55) | 23 (-) | 16 (70) | 7 (30) |
| Ventricular CSF at surgery, n (%) | 112 (77) | 45 (40) | 67 (60) | 18 (78) | 13 (72) | 5 (28) |
| Ventricular CSF at follow-up, n (%) | 34 (23) | 20 (59) | 14 (41) | 5 (22) | 3 (60) | 2 (40) |
| Spearman’s rho, median (IQR) | 0.6 (0.5) | 0.5 (0.7) | 0.6 (0.3) | - | - | - |

**Table** S1 Overview of results. Left side of the table lists all proteins detected in the indicated compartment, while the right side of the table lists the number of proteins with a *p*-value ≤ 0.05. The numbers show the amount of proteins with a higher level in respective compartment.
